# Module-based multiscale simulation of angiogenesis in skeletal muscle

**DOI:** 10.1186/1742-4682-8-6

**Published:** 2011-04-04

**Authors:** Gang Liu, Amina A Qutub, Prakash Vempati, Feilim Mac Gabhann, Aleksander S Popel

**Affiliations:** 1 Systems Biology Laboratory, Department of Biomedical Engineering, School of Medicine, Johns Hopkins University, Baltimore, MD 21205, USA; 2 Department of Bioengineering, Rice University, Houston, TX 77521, USA; 3 Institute for Computational Medicine and Department of Biomedical Engineering, Johns Hopkins University, Baltimore, MD 21218, USA

## Abstract

**Background:**

Mathematical modeling of angiogenesis has been gaining momentum as a means to shed new light on the biological complexity underlying blood vessel growth. A variety of computational models have been developed, each focusing on different aspects of the angiogenesis process and occurring at different biological scales, ranging from the molecular to the tissue levels. Integration of models at different scales is a challenging and currently unsolved problem.

**Results:**

We present an object-oriented module-based computational integration strategy to build a multiscale model of angiogenesis that links currently available models. As an example case, we use this approach to integrate modules representing microvascular blood flow, oxygen transport, vascular endothelial growth factor transport and endothelial cell behavior (sensing, migration and proliferation). Modeling methodologies in these modules include algebraic equations, partial differential equations and agent-based models with complex logical rules. We apply this integrated model to simulate exercise-induced angiogenesis in skeletal muscle. The simulation results compare capillary growth patterns between different exercise conditions for a single bout of exercise. Results demonstrate how the computational infrastructure can effectively integrate multiple modules by coordinating their connectivity and data exchange. Model parameterization offers simulation flexibility and a platform for performing sensitivity analysis.

**Conclusions:**

This systems biology strategy can be applied to larger scale integration of computational models of angiogenesis in skeletal muscle, or other complex processes in other tissues under physiological and pathological conditions.

## Background

Angiogenesis is a complex process whereby new capillaries are formed from pre-existing microvasculature. It plays important roles in many physiological processes including embryonic development, wound healing and exercise-induced vascular adaptation. In such processes, robust control of capillary growth leads to new healthy pattern of physiological vessel network that matches the metabolic demands of development, wound repair, or exercise [1]. In contrast, excessive or insufficient growth of blood vessels is associated with an array of pathophysiological processes and diseases, among which are malignant tumor growth, peripheral artery disease, diabetic retinopathy, and rheumatoid arthritis [1].

Systems-level studies of angiogenesis in physiological and pathophysiological conditions improve our quantitative understanding of the process and hence aid in therapeutic design. Extensive experimental studies of angiogenesis over the past two decades have revealed that the angiogenesis process is comprised of a series of events at multiple biological organization levels from molecules to cells, tissues, and organs. For example, as a first approximation, exercise-induced angiogenesis can be described as a sequence of the following events: i) Exercise increases oxygen consumption in tissue, followed by increased blood flow in the vasculature, thus affecting convection-diffusion oxygen transport processes [2]; ii) As exercise continues, insufficient oxygen delivery to the tissue leads to tissue cellular hypoxia, which results in activation of the transcription factor hypoxia-inducible factor 1α (HIF1α) [3] and the transcription coactivator peroxisome-proliferator-activated-receptor-gamma coactivator 1α (PGC1α) [4]; iii) These factors induce the upregulation of vascular endothelial growth factor (VEGF) expression [5]. VEGF is secreted from myocytes (and possibly stromal cells), diffuses through the interstitial space, and binds to VEGF receptors (VEGFRs) on microvascular endothelial cells; concomitantly, endothelial cell expression of VEGFRs is also altered [6]; iv) The increase in VEGF and VEGFR concentration and possibly VEGF gradients results in activation of endothelial cells and cause capillary sprouting. Thus new capillaries and anastomoses form and new capillary network patterns develop [7]; v) After exercise, VEGF and VEGFR expression remain elevated for a limited time and thereafter return to basal levels [6]. The signaling set in motion causes blood vessel remodeling to continue after exercise. Thus, the time scales of individual events range from seconds in oxygen convection-diffusion processes to hours in VEGF reaction-diffusion processes, to days or weeks in capillary sprouting processes. Spatial scales vary from nanometers at the molecular level to microns at the cellular level, to millimetres or centimetres at the tissue level.

The complexity of angiogenesis is a function not only of the multiscale characteristics in temporal and spatial domains, but also of the combinatorial interactions between key biological components across organizational levels. At the molecular level, multiple HIF-associated molecules and hundreds of genes activated by HIF form a complex transcriptional regulatory network [8]. Six isoforms of VEGF-A (VEGF_121_,_145_,_165_,_183_,_189_,_206_), three VEGFRs (VEGFR-1, -2, -3) and two coreceptors (neuropilin-1 and -2) constitute a complex ligand-receptor interaction network, regulating intracellular signaling and determining cellular response [9]. In addition, other VEGF proteins, like placental growth factor (PlGF) and VEGF-B, -C, -D, compete with VEGF-A for some of the same receptors. Matrix metalloproteinases (MMPs) also form a key molecular family with approximately 30 members; MMPs are capable of proteolyzing components of the extracellular matrix (ECM) thus decreasing the physical barriers encountered by a tip endothelial cell leading a nascent capillary sprout [10]. At the cellular level, endothelial cell activation, migration and proliferation are driven by local growth factor concentrations and gradients. Capillary sprouting is also governed by the interaction between a tip cell and its following stalk cells, and by cell adhesion to the ECM [11]. In addition, parenchymal cells, precursor cells and stromal cells, as well as the ECM, constitute the nascent sprout microenvironment, influencing endothelial cell signaling, adhesion, proliferation and migration.

Mathematical and computational models of angiogenesis have become useful tools to represent this level of biological complexity and shed new light on key control mechanisms. In particular, computational modeling of tumor-induced angiogenesis has been an active area of research over the past two decades and has also been extensively reviewed [12-15]. Here we give a brief overview of the angiogenesis models relevant to building a multiscale model of angiogenesis in skeletal muscle using different modeling methodologies. The models can be classified into continuous, discrete and hybrid categories. Continuum models of growth factor activity often applied molecular-detailed reaction and reaction-diffusion differential equations. These models have been used to describe many aspects of angiogenesis, e.g., host tissue distribution of a chemotactic factor following its secretion from a tumor [16], VEGF-VEGFR interactions [17], a fibroblast growth factor-binding network [18], whole-body compartmental distribution of VEGF under exercise and peripheral artery disease conditions [19,20], the contribution of endothelial progenitor cells to circulation of VEGF in organs and their effects on tumor growth and angiogenesis [21], and a VEGF reaction-transport model in skeletal muscle [22]. Models of other angiogenesis-associated proteins such as MMP2 and MMP9 also have been developed [23,24]. By describing capillary networks in terms of endothelial cell densities, continuum models have also been developed to represent tumor-induced capillary growth [25-27] and the wound healing process [28]. Discrete models such as cellular automata [29], cellular-Potts model [30], and agent-based models [31-33] have been developed to describe tissue behavior stemming from the interaction between cells, extracellular proteins and the microenvironment. These cell-based models offer unique capability of representing and interpreting blood vessel growth pattern as an emergent property of the interactions of many individual cells and their local microenvironment. By combining the continuum approach with the cell-based modeling approach, hybrid modeling can be used to describe the *in vivo *vascular structure along with detailed molecular distributions [34-37], providing appropriate computational resolution across various scales.

With a number of computational models currently available to describe different aspects of angiogenesis, integration of existing models along with new biological information is a promising strategy to build a complex multiscale model [38,39]. While current advances mainly focus on the representation format of molecular interaction models (e.g., XML-based notation) and dynamic integration of these models (e.g., Cytosolve [40]), few strategies exist to combine existing models at multiple scales with mixed methodologies. Here we describe our development of a novel computational infrastructure to coordinate and integrate modules of angiogenesis across various scales of biological organization and spatial resolution. Using this approach can significantly reduce model development time and avoid repetitive development efforts. These modules can be adapted from previously-developed mathematical models. Our laboratory has developed a number of angiogenesis models including: oxygen transport [41]; VEGF reaction-diffusion [22]; capillary sprouting [33]; FGF-FGFR ligand-receptor binding kinetics [18]; MMP proteolysis [23,24,42]; and MMP-mediated VEGF release from the ECM [43]. We show results of a test case, which integrated a blood flow model, an oxygen transport model, a VEGF transport model and a cell-based capillary sprouting model. With the use of Java Native Interface functions, previously-developed angiogenesis models were redesigned as "pluggable modules" and integrated into the angiogenesis modeling environment. Another advantage of this simulation infrastructure is its flexibility, allowing integration of models written using different simulation techniques and different programming languages. Note that the primary aim of this study is building methodology for multiscale modeling, rather than obtaining novel physiological results; detailed simulations of skeletal muscle angiogenesis and comparison to experimental data will be presented elsewhere.

The computational scheme presented here fits into the Physiome Project defined as a computational framework allowing the integration of models and databases that intends to enhance the descriptive, integrative and quantitative understanding of the functions of cells, tissues and organs in human body [44-46]. Integral parts of the Physiome Project are the Cardiac Physiome [47], the Microcirculaton Physiome [48,49], and the EuHeart project http://www.euheart.eu/, which are aimed at specific organs or physiological systems. The Virtual Physiological Human project is also aimed at a quantitative description of the entire human [50-52]. To achieve the goals of these projects, it is essential to share computational models between a variety of modeling methodologies, computational platforms, and computer languages and incorporate them into integrative models. One approach in the past decade is to develop XML-based markup languages to facilitate model representation and exchange. The two most-accepted formats, SBML [53] and CellML [54], are designed to describe biochemical reaction networks in compartmental systems expressed by ordinary differential equations (i.e., they have no spatial description). FieldML [55] allowing for spatial description is under development. Alternatively, the object-oriented modeling methodology provides a strategy to describe the biological organizations and flexible solution to integrate currently available models. For example, universal modeling language (UML) [56,57] and other meta-languages such as E-cell [58] have been proposed. However, the robustness of the integration of external models is dependent on the interface of these meta-languages. In the current study, we propose to use a natural object-oriented language, Java, as a modeling language to design the integration controller and link currently available modules at different scales.

## Systems and Methods

**Figure 1:**
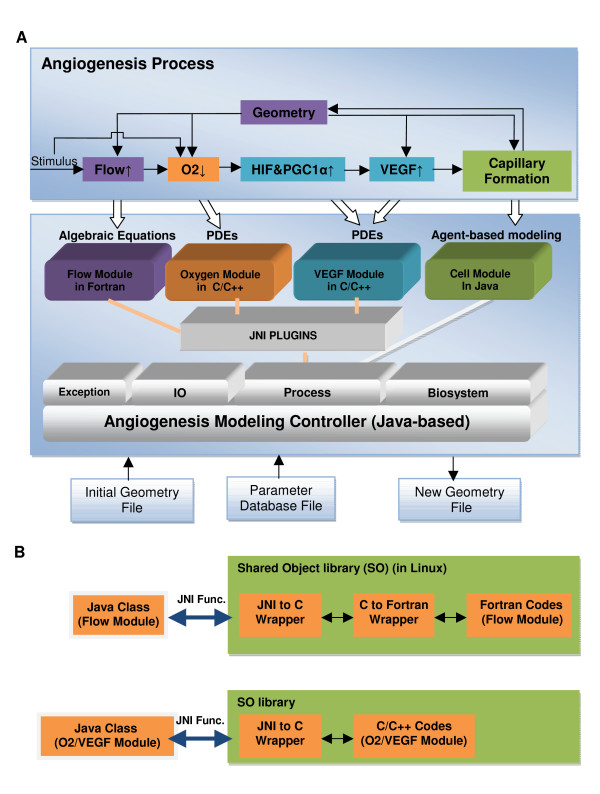
**Schematics of Module-based Mulitscale Angiogenesis Modeling Methodology**. **A) **Skeletal muscle angiogenesis is modeled as a multi-step process. It starts with a blood flow simulation followed by a simulation of oxygen convection-transport process. Using O_2 _tissue distribution, VEGF secretion by myocytes is computed as a function of oxygen-dependent transcription factors HIF1α and PGC1α; then a VEGF reaction-transport process is computed. Lastly, capillary formation is simulated based on VEGF concentration and gradients. Feedback loops increase the complexity of the model since a new geometry with nascent vessels will affect blood flow conditions, tissue hypoxia, and VEGF secretion and distributions. All four processes are simulated using a variety of modeling techniques and languages. We use Java as the language for modeling the controller, and apply JNI plugins to link these modules together. The controller is composed of four sub-packages, including Process, Biosystems, IO and Exceptions. **B) **Communications between different modules and Java codes in core package are implemented by transferring each module into a shared object library (SO file in Linux). Upper panel shows that two wrapper files (includes Java-to-C and C-to-Fortran wrapper) are written to communicate between the flow Java class defined in the controller and the Fortran flow module, to call the flow module in Fortran. Lower panel shows that a JNI C wrapper is required to transfer the data between the modeling controller (in Java) and the Oxygen/VEGF module (in C/C++).

We describe a computational platform capable of linking any number of modules. In the particular example of skeletal muscle angiogenesis, we integrate four modules: microvascular blood flow; oxygen transport; VEGF ligand-receptor interactions and transport; and a cell module describing capillary sprout formation. These four modules use diverse modeling methodologies: algebraic equations (blood flow), partial differential equations (PDEs, oxygen and VEGF transport) and agent-based modeling (ABM, cell model). An overview of the simulation scheme is shown in Figure 1A. Briefly, the model initiates with the input of a three-dimensional (3D) muscle tissue geometry that includes muscle fibers and a microvascular network; rat extensor digitorum longus (EDL) muscle is used as a prototype, as previously described [22]. This tissue geometry is first used to calculate blood flow in the vascular network, and then in the computation of oxygen distribution in the vascular and extravascular space, followed by simulation of VEGF distribution in the interstitial space and on the endothelial surface, and finally simulation of capillary sprouting and remodeling of the vascular network. Blood flow and hematocrit are simulated using the two-phase continuum model proposed by Pries *et al *[59]. Oxygen transport model [60] is used to calculate the spatial distribution of oxygen tension throughout the tissue. VEGF secretion from myocytes through an oxygen-dependent pathway is described by an experiment-based oxygen-dependent transfer function dependent on the factors HIF1α and PGC1α (details are below). A modified VEGF reaction-diffusion model [22,61] is used to predict the spatial VEGF distribution in tissue interstitial space and at the surface of the endothelium. Using our agent-based model with this VEGF concentration profile defined as input [33], we further compute elongation, proliferation and migration of endothelial cells forming capillary sprouts. The result is a new capillary network. In turn, this new structure feeds back into the integrated model as an updated vascular geometry, and starts a new cycle with the flow model, oxygen transport model and VEGF reaction-diffusion model, thus simulating the dynamics of the angiogenesis process. Governing equations and a brief description of each individual module are given below.

### Skeletal Muscle Tissue Geometry

A 3D representation of muscle tissue structure is constructed using a previously-described algorithm [22]; it includes cylindrical fibers arranged in regular arrays and a network of capillaries, small precapillary arterioles and postcapillary venules. The dimensions of the tissue studied are 200 μm width (x-axis), 208 μm height (y-axis) and 800 μm length (z-axis). The fiber and vascular geometry can be specified using different methods, including tissue-specific geometries with irregular-shaped fibers obtained from *in vivo *imaging; tissue dimensions can also be extended.

### Flow Module

The *in vivo *hemorheological model [59,60] is applied to calculate the distribution of blood flow rate (*Q*) and discharge hematocrit (*H_D_*), among all the capillary segments under steady state conditions during exercise. The governing equations are derived from the mass conservation law for volumetric blood flow rate and red blood cell flow rate at the *j^th^*node (vascular bifurcation), as follows:(1)(2)

Here *Q_ij _*is the volumetric flow rate in vessel segment *ij*, a cylinder whose ends are the *i^th^*and *j^th^*nodes of the network. Flow rate is calculated as *Q_ij _*= π*R*^4^(*p_i_*-*p_j_*)/(8*ηL*), where *p*_*j *_is the hydrodynamic pressure at node *j*, *R *and *L *are the radius and length of the segment, and *η *is the apparent viscosity which is a function of *R *and *H*_*D *_(Fahraeus-Lindqvist effect). These equations are supplemented by the empirical equations governing red blood cell-plasma separation at vascular bifurcations. The system of nonlinear algebraic equations for all *N *segments is solved with respect to pressure and discharge hematocrit, from which flow in each segment is calculated.

### Oxygen Module

Oxygen delivery from the microvasculature to skeletal muscle myocytes is one of the key functions of microcirculation. During exercise, oxygen consumption may increase many folds compared to resting state, affecting both extravascular and intravascular oxygen transport. The oxygen model consists of two partial differential equations, Eqns. 3 and 4, governing extravascular and intravascular oxygen transport, respectively, assuming muscle fibers and interstitial space are a single tissue phase [41,60].

Oxygen tension in the tissue, *P_O2 _*= *P*(x,y,z,t), is governed by free oxygen diffusion, myoglobin-facilitated diffusion, and oxygen consumption by tissue cells:(3)

Here  and *D_Mb _*are the diffusivities of oxygen and myoglobin in tissue respectively; *S_Mb _*is the oxygen-myoglobin saturation; *α_tis _*is the oxygen solubility in tissue;  is the binding capacity of myoglobin with oxygen; *M*_*c *_is the oxygen consumption rate coefficient for Michaelis-Menten kinetics; *P_crit _*is the critical *P_O2 _*at which oxygen consumption equals to 50% of *M_c_*; and *S*_*Mb *_is defined as *P*/(*P*+*P_50,Mb_*) assuming the local binding equilibrium between oxygen and myoglobin, where *P_50,Mb _*is the *P_O2 _*necessary for 50% myoglobin oxygen saturation.

Oxygen transport in the blood vessels is governed by:(4)

Here  is the oxygen-hemoglobin saturation in blood vessel; *α_RBC _*and *α_pl _*are oxygen solubility in red blood cell and plasma, respectively; *P_b _*is the oxygen tension in blood plasma; *ν_b _*is the mean blood velocity (*ν_b _*= *Q*/(π*R*^2^)); *H_T _*and *H_D _*are the tube and discharge hematocrit, calculated from blood flow model;  is binding capacity of hemoglobin with oxygen; *ξ*is the distance along a vessel's longitudinal axis; *J_wall _*is the capillary wall flux; and  is defined as  assuming the binding equilibrium between oxygen and hemoglobin, where *P_50,Hb _*is the *P_O2 _*necessary for 50% hemoglobin oxygen saturation.

In addition, continuity of oxygen flux at the interface between blood vessels and tissue yields:(5)

where *n *is the unit normal vector, *P_wall _*is the local *P_O2 _*at the vessel wall and *k_0 _*is the mass transfer coefficient estimated from an empirical equation *k_0 _*= 3.15+3.26*H_T_*-9.71+9.74(*H_T_*)^2 ^+ 8.54()^2^. The system of nonlinear partial differential equations was solved using the finite difference method, with a grid size of 1 micron as described in [60].

### VEGF module

VEGF is the most-studied molecular factor involved in angiogenesis, including exercise-induced angiogenesis. Among several splice isoforms in the VEGF family, VEGF_120 _and VEGF_164 _(in rodents; human isoforms are VEGF_121 _and VEGF_165_) are considered to be the major pro-angiogenic cytokines that induce proliferation and migration of endothelial cells. The molecular weights of VEGF_164 _and VEGF_120 _are 45 and 36 kDa respectively and thus their diffusion coefficients are slightly different; in addition, VEGF_164 _binds the heparan sulfate proteoglycans (HSPGs) while VEGF_120 _does not and thus the shorter isoform diffuses more freely through the ECM.

A reaction-diffusion model [22] is used to predict molecular distribution in the interstitial space and on the endothelial surface. The governing equations for VEGF_164 _and VEGF_120 _are:(6)(7)

where *C*_*V164*_, C_*V120 *_, *C_H _*and C_*V*164•*H *_are the concentrations of VEGF_164_, VEGF_120_, HSPG and VEGF_164_·HSPG complex; *D_V164 _*and *D_V120 _*are the diffusion coefficients of VEGF_164 _and VEGF_120_; and *k_on,V164,H _*and *k_off,V164,H _*are the association and dissociation rate constants between VEGF_164 _and HSPG. The boundary conditions for VEGF_164 _and VEGF_120 _at the surfaces of muscle fibers and endothelial cells, and the complete details of ligand-receptor interactions, were described in [61].

The model describes the secretion of two VEGF isoforms from the muscle fibers, molecular transport of each isoform in the interstitial space, binding of VEGF_164 _to HSPG in the ECM, VEGF_164/120 _binding to VEGFR2 at the endothelial cell surface and internalization of these ligand-receptor complexes. The model also considers VEGFR1 and neuropilin-1 (NRP1) coreceptor binding with VEGF ligands.

We previously applied an empirical equation to describe the relationship between *P_O2 _*and VEGF secretion rate to estimate local fiber VEGF secretion [22]. The empirical relationship was derived by combining experimentally-based relationships between intracellular HIF1α concentration and *P_O2 _in vitro*, and HIF1α concentration and VEGF secretion *in vivo *in skeletal muscle. However, PGC1α has recently been found to be another important regulator of VEGF in exercise [4,62]. It was first discovered as a cold-inducible transcriptional coactivator for nuclear hormone receptors in brown fat and an enhancer of mitochondrial metabolism and function [63,64]. Recently, a series of experimental studies [65-67] have shown that VEGF gene expression and protein levels are highly dependent on the presence and concentration of PGC1α, through HIF-independent and HIF-dependent pathways. Thus, we have modified the equation by incorporating the effect of PGC1α:(8)

Here *S*_*VEGF *_is the VEGF secretion rate, *S_B,VEGF _*is basal secretion rate at normoxic levels of [HIF1α]. It is defined as a function of [PGC1α], written as a sigmoidal form, , where [PGC1α] is the PGC1α concentration normalized relative to normoxic expression for wild type skeletal muscle, *n_p _*is the Hill constant, and *k_h _*, A, and B are empirical constants. *S_0,VEGF _*is defined as basal VEGF secretion rate at normoxic levels of [HIF1α] and [PGC1α] for wild type skeletal muscle. Equations for oxygen-dependent [PGC1α] under wild type, knockout and over-expression conditions are shown in additional file 1 (Eqns. S4-S6). Data fitting based on an array of experimental data [4,66,67] results in the following parameters: A = 2.3167, B = 0.35, *k_h _*= 2.5641, *n_p _*= 1.086,  = 3.

### Cell Module

The Cell module is adapted from our 3D agent-based *in vitro *model [33] to describe how capillary endothelial cells respond to stimuli, specifically VEGF concentration and gradients, during the time course of sprout formation. The model applies logical rules to define cell activation, elongation, migration and proliferation events, based on extensive published experimental data [33]. The model makes predictions of how single-cell events contribute to vessel formation and patterns through the interaction of various cell types and their microenvironment.

The primary rules used in the *in vitro *model [33] are specified as follows: i) Endothelial cells are activated at an initial time point and the number of activated cells is constrained by a specified maximum number per unit capillary length. This activation initiates development of a tip cell segment of a sprout, later followed by the formation of a first stalk cell segment. ii) Following invasion of new sprouts into the tissue by extending the leading tip and stalk cell segments, the tip cell continues to migrate in the interstitial space following VEGF gradients and moves towards higher VEGF concentration. In addition, the tip cell can also proliferate with a certain probability, and the stalk cell can elongate and proliferate in a specified fashion; note that the probability of tip cell proliferation is much smaller than that of the stalk cells. The combined effect of these two cell phenotypes can be simulated as a biophysical push-pull system. iii) Branching occurs with a specified probability after a designated time threshold has elapsed at either a stalk or tip cell. The branching angle is selected stochastically and is less than 120 degrees. In the original model [33] the frequency of branching events during the spouting process was a function of the expression of ligand Dll4 and receptor Notch on the endothelial cells. Details of other rules and the parameters were described in [33].

To simulate *in vivo *conditions in the skeletal muscle vessel network, we modified some of the previously-defined rules and introduced additional rules to the model. Since muscle fibers and vasculature occupy respectively 79.7% and 2.5% of the tissue volume, the interstitial space totals 17.8%. Hence the freedom of tip endothelial cell migration during sprout formation is constrained to occur in a small volume of interstitial space. Note that in the model the endothelial cells consist of cylindrical cell segments (10 μm length and 6 μm diameter per capillary segment; 4 segments per cell defined in this study); the rules are formulated for these segments rather than for whole cells. This part of the model can be readily modified. The additional rules imposed in this study are as follows: i) Elongation or migration of cell segments follows the original rules as developed and defined in [33], except when the cell may encounter a fiber by following the growth factor gradient, we assume that the tip cell filopodia will sense the fiber and instead the cell follows the second largest VEGF gradient direction alternatively to elongate or migrate. ii) Anastomoses are formed when the tip cell senses an existing capillary or a sprout within 5 microns. iii) Since the function of Dll4-Notch is not clearly defined in skeletal muscle, their effects are not taken into account in the current simulations, but this effect can be readily added. For model simplification and demonstration purpose, tip cell elongation and the branching are not allowed in the present study.

### Integration of computational modules

The development of an anatomically-, biophysically- and molecular-detailed spatio-temporal model by integration of different modules is a novel and challenging task. One of the main objectives of this study is to create a platform for integration of different modules written in different programming languages and using mixed modeling methodologies. The component modules may be created in the same or different laboratories, and could also be selected from a public model database. The difficulty of this task stems from the fact that few standards and open-source software/libraries for PDE solvers and ABMs exist. As a result, modules are dependent on their native languages and on differential equation solvers, making the integration difficult. Another problem facing the integration of modules is how to define and implement the connectivity between them, i.e., the exchange of data between the modules. Here we solve these two problems using a novel computational infrastructure and object-oriented design as described below.

### Computational Infrastructure

To overcome the language barrier between the four modules selected in this study (Fortran for the Flow module, C/C++ for the Oxygen and VEGF modules and Java for Cell Module, Figure 1A), we choose Java to design the controller, which provides a flexible high-level interface and object-oriented facilities. Instead of rewriting the codes in each module in Java, we use a mixed-language programming environment to link the modules and save repetitive effort. The native codes in Fortran and C run faster than Java, and this compromise solution can also inherit advantages of these two languages. Another important technical aspect that renders this hybrid system feasible is the existence of Java Native Interface (JNI) API to convert functions and data type from native codes (Fortran and C) automatically to Java. To fulfil this purpose, we redesigned the native codes for the Flow, Oxygen, and VEGF modules to the format of functions and subroutines, and compiled these codes into the Java-readable libraries, turning all four modules into "pluggable" libraries that can be called by the controller coded in Java (Figure 1B). Furthermore, these libraries can be dynamically linked, making the simulation of dynamic angiogenesis processes feasible. Thus, using the controller as a bridge between each module, communication between different modules is relatively easy to implement. Last, it is easy to use Java to implement the connection between core codes and a new parameter database file used by the four selected modules.

To achieve high performance of native codes, parallel computing is implemented in the Oxygen and VEGF modules, as they require extensive computing resources. The current version of the modules adds OPENMP (open multi-processing) support, an industry standard for memory-shared parallel systems, to shorten the simulation time when the PDE solver is called by the controller. Numerical simulation time for the Oxygen and VEGF PDEs has been sped up ~5 fold using an 8 quad-core processor.

### Object-oriented design

As a starting point to integrate all four modules, we focused on robust design of the controller, providing the connectivity between the modules, rather than providing solvers/software for mathematical models (i.e., rather than focusing on the capability of solving equations or agent-based models numerically). This will not impose constraints on the modeling methodology used in the modules, and it will allow a wide choice of modules to be integrated, providing more flexibility. More detail on the procedure for integration of modules at multiple time scales using object-oriented classes is given below in the section "Integration of modules at multiple time scales".

**Figure 2:**
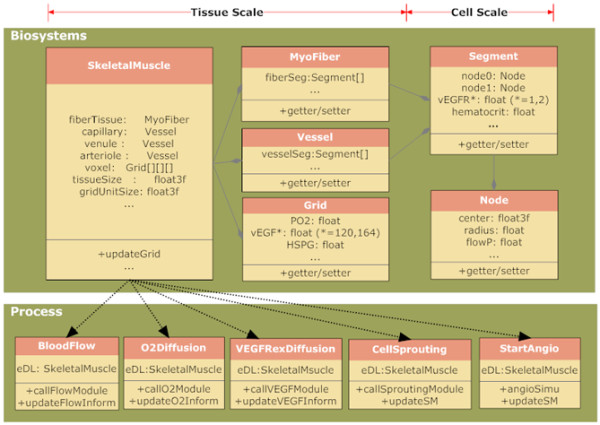
**Object-oriented design for the angiogenesis modeling package**. Major classes across tissue and cell scales in the modeling controller are shown. They include SkeletalMuscle, Myofiber, Vessel, Grid, Segment and Node classes in the Biosystems subpackage, and BloodFlow, O2Diffusion, VEGFRxnDiffusion, CellSprouting, and StartAngio classes in the Process subpackage. The hierarchical structure of relationships between the classes is represented by arrows.

Regarding design at the upper level (i.e., package design), we devised four sub-systems: Biosystem, Process, IO (Input/Output) and Exception as shown in lower panel in Figure 1A. The Biosystem is a repository of hierarchical structure of biological and biophysical information in the tissue as shown in Figure 2. The Process is composed of angiogenesis subprocesses occurring at various stages in growth process. The IO is used to provide an interface for the interaction between the system and the user, for example, to read parameters used for the simulation of the Oxygen and VEGF modules. The Exception provides the capability to debug and handle runtime errors.

At the lower level (i.e., class design), we applied object-oriented concepts to describe a hierarchical structure of skeletal muscle (EDL) and events in the angiogenesis process. The class diagram shown in Figure 2 depicts the relationships among the major classes proposed. In the Biosystem sub-package, six classes are defined to describe the entities composing the tissue of interest: SkeletalMuscle, Myofiber, Vessel, Grid, Node and Segment. In addition, angiogenesis event classes defined in the sub-package Process include BloodFlow, O2Diffusion, VEGFRxnDiffusion, CellSprouting and StartAngio.

The Node and Segment classes in the Biosystem sub-package are defined below the cell scale. The Node class represents the circular surface of cylindrical segments at their ends. It contains spatial information, including the circle center position and the circle radius. It may also have biophysical information such as blood pressure, flow velocity and hematocrit if the node is contained in a blood vessel. The Segment class represents a cylinder in 3D space, corresponding to either a fraction of a blood vessel or muscle fiber (assuming the fiber and blood vessel are cylindrical in shape). A Segment object contains two Node objects at its ends and the length of the cylinder. Each Segment also contains biophysical information such as blood flow, pressure and hematocrit, and VEGF receptor density if the segment type is a blood vessel.

At the tissue scale, SkeletalMuscle, Myofiber, Vessel and Grid are defined in the Biosystem sub-package. The Vessel class is used for representation of microvessels including capillaries, venules and arterioles. The Myofiber class is used for representation of skeletal muscle myocytes. These two classes are each composed of a series of segments. Interstitial space information including VEGF and HSPG concentrations is described in the Grid class. The Grid class also contains the local *P_O2 _*value. Ensemble components of fiberTissue, capillary, venule, arteriole, voxel, tissueSize and gridUnitSize render the SkeletalMuscle class, which describes detailed skeletal muscle structure.

In the Process sub-package, four classes including BloodFlow, O2Diffusion, VEGFRxnDiffusion and CellSprouting provide connections with Java-readable libraries compiled from their corresponding modules. The calling of these Java classes involves three steps: i) The skeletal muscle object (realization of SkeletalMuscle class) will be initialized and then transferred as the input to each module. ii) Each specific module will compute using their intrinsic numerical solvers and then the results will be transferred to the Java interface class. iii) Finally, the skeletal muscle object will be updated with the solutions. The StartAngio class contains methods defined to specifically simulate exercise-induced angiogenesis.

### Developing the computational environment

The simulation experiments were run on a computer with 64 bit Linux Ubuntu system, 8 quad-CPU and 128 Gbyte memory. Eclipse http://www.eclipse.org is used as an Integrated Development Environment for coding purposes. JDK (Java Development Kit) 1.6.16 (Oracle, Redwood Shores, CA) is used as Java compiler, and Intel Fortran/C++ compiler suite (v.11.1) (Intel, Santa Clara, CA) is used as Fortran/C++ compiler. We also incorporate Java 3D™ API (Oracle, Redwood Shores, CA) for 3D programming purposes and the Log4j package http://logging.apache.org/log4j/1.2 to log all the runtime messages for the purpose of debugging. We use Bazaar http://bazaar.canonical.com as our source code version control system since as an industry standard for software development it provides support for a large scale project development by a team of programmers. It has advantages in terms of branching, merging and keeping revision versions. The GNU Make tool http://www.gnu.org/software/make/ is chosen for automation of building executable programs and libraries from source codes, and running programs from binary codes. In particular, it is useful for a hybrid system (i.e., mixed programming environment) in a single program. The MASON package http://cs.gmu.edu/~eclab/projects/mason/ is used as the agent-based modeling library. Unit tests are performed using the JUNIT 4.0 package http://www.junit.org/.

### Integration of modules at multiple time scales

We performed the integration of the modules using a sequential method, that is, the modules are run sequentially rather than in parallel. This is based on a time scale analysis of each process integrated into the multiscale angiogenesis model. We computed the module at the fastest time scale first. The outcome of that module was considered as a pseudo-steady state and used as an input feeding into the modules at slower time scales. This continued sequentially until we computed the module at the slowest time scale. Specifically, the blood flow regulation and oxygen distribution modules reach equilibrium within seconds to minutes; VEGF gradients at time scales of minutes to hours; and capillary sprouting from hours to weeks. Thus we computed the steady-state flow and oxygen module first, then used their simulation results as an input to VEGF reaction-diffusion model and run PDE solver to compute VEGF profile, and then finally run the agent-based model to simulate angiogenesis patterns for single-bout exercise. When endurance exercise for days or weeks is simulated, the updated model geometry will be used as the new input to run flow, oxygen and cell modules sequentially.

### Example run of a simulation of single-bout exercise

Using the object-oriented design concept, we constructed a controller which is capable of interacting with each individual module defined in our multiscale model. A run of the simulation starts with input of geometry files and parameter database file (written in database file format). These inputs initialize an object of "SkeletalMuscle" class with the 3D coordinate information of segments and nodes for skeletal muscle fiber and blood vessel network. The parameter database file assigns values to the biochemical and physiological parameters defined in the model. Following this, the controller calls the flow module as a dynamic linked library (dll) as described above, using the geometric and biochemical parameters as input. The flow module has its own built-in solver that returns (to the controller) results including blood pressure, hematocrit, viscosity and flow rates in the various vessel segments. The information is stored in the controller "SkeletalMuscle" object. This object, now with the updated flow information and the increased oxygen consumption rate, is passed by the controller to the oxygen module. This module computes the oxygen tension at each grid point defined within the skeletal muscle and passes the values back to the controller to be stored in the voxel field (Grid class) of the "SkeletalMuscle" object (element *P_O2 _*is defined in Grid Class). The controller passes the O_2_-updated object to the VEGF module, which includes O_2_-PGC1-HIF-VEGF empirical equations. Upon completing its simulation, the VEGF module passes the VEGF, HSPG, and other concentrations throughout the tissue back to the controller. This spatial concentration profile information is then passed to the cell module to simulate capillary growth. During the 8-hour post-exercise period, due to the cessation of exercise, blood flow rate and oxygen consumption rate return to basal level. We thus assume that capillary sprouting is the only active module during this period; blood flow rate and oxygen distributions are assumed to be at basal levels.

## Results

Using the integration strategy and simulation package described above, we performed a series of computational experiments to simulate activity-induced angiogenesis in EDL during a single bout of exercise. First, a 3D simulation was conducted to sequentially integrate modules from blood flow to oxygen convection-diffusion, to VEGF diffusion-reaction, to capillary sprouting. Second, sensitivity analysis was used to examine sensitivities of key biochemical, biophysical and physiological parameters to capillary growth. Third, angiogenesis patterns were compared between different exercise intensities, and high- or low-inspired oxygen conditions.

### Sequential integration of modules from blood flow to capillary sprouting

Single-bout exercise was simulated by dynamic integration of four modules: Flow, Oxygen, VEGF and Cell. The simulations are based primarily on physiological parameters used in our previously developed models [33,60,61], as shown in Table 1. Most of the parameters were taken from experimental data for rat skeletal muscle while others were adopted from measurements in other tissues or theoretical estimates when parameters are unavailable from literature (e.g., diffusion coefficients for VEGF_164/120_, secretion rates for VEGF_164/120_). Here we describe several key parameters used in the simulation.

**Table 1:** Parameters of the multiscale model*

Parameter	Description	Value	Unit	Module
*P_in_*	Inlet pressure	10	mmHg	Flow
*H_d,in_*	Inlet hematocrit	0.4	-	Flow
*α_tis_*	O_2 _solubility in tissue	3.89 × 10^-5^	ml O_2 _ml^-1 ^mmHg^-1^	Oxygen
*α_RBC _*	O_2 _solubility inside RBC	3.38 × 10^-5^	ml O_2 _ml^-1 ^mmHg^-1^	Oxygen
*α_pl _*	O_2 _solubility in plasma	2.82 × 10^-5^	ml O_2 _ml^-1 ^mmHg^-1^	Oxygen
*D_O2_*	O_2 _diffusivity in tissue	2.41 × 10^-5^	cm^2 ^s^-1^	Oxygen
*D_Mb_*	Myoglobin diffusivity in tissue	1.73 × 10^-7^	cm^2 ^s^-1^	Oxygen
*M_c_*	Mass consumption O_2 _by tissue	1.5 × 10^-3^, 1.67 × 10^-4^	ml O_2 _ml^-1 ^s^-1^	Oxygen
	Myoglobin O_2_-binding capacity	1.016 × 10^-2^	ml O_2 _(ml tissue)^-1^	Oxygen
	Hemoglobin O_2_-binding capacity	0.52	ml O_2 _(dl RBC)^-1^	Oxygen
*P_50,Mb_*	*P_O2 _*for 50% myoglobin oxygen saturation	5.3	mmHg	Oxygen
*P_50,Hb_*	*P_O2 _*for 50% hemoglobin oxygen saturation	37	mmHg	Oxygen
*n_h_*	Oxyhemoglobin saturation Hill coefficients	2.7	-	Oxygen
*S_O2A_*	Oxygen saturation for arteriolar inlets	0.6,0.3	-	Oxygen
*D_V120_*	V_120 _diffusivity in ECM	1.13 × 10^-6^	cm^2 ^s^-1^	VEGF
*D_V164_*	V_164 _diffusivity in ECM	1.04 × 10^-6^	cm^2 ^s^-1^	VEGF
*H_ECM_*	HSPG density in ECM	7.5 × 10^-10^	pmol μm^-3 ^ECM	VEGF
*k_on,v164,H_*	V_164_HSPG complex association rate constant	4.2 × 10^8^	pmol^-1 ^μm^3 ^s^-1^	VEGF
*K_off,v164,H_*	V_164_HSPG complex dissociation rate constant	1 × 10^-2^	s^-1^	VEGF
*k_int_*	R1 and -R2 internalization rate (free and complex form)	2.8 × 10^-4^	s^-1^	VEGF
*S_R_*	R1 and -R2 insertion rate	9.2 × 10^-16^	pmol μm^-2 ^s^-1^	VEGF
*H_EBM_,H_MBM_*	HSPG density in EBM and MBM	1.3 × 10^-8^	pmol μm^-3 ^BM	VEGF
*k_on,vegf,R1_*	V_120_R1 (V_164_R1 ) complex association rate constant	3 × 10^10^	pmol^-1 ^μm^3 ^s^-1^	VEGF
*k_on,vegf,R2_*	V_120_R2 (V_164_R2 ) complex association rate constant	1 × 10^10^	pmol^-1 ^μm^3 ^s^-1^	VEGF
*k_off,vegf,R1,_k_off,vegf,R2_*	V_120_R1/R2, V_164_R1/R2 complex dissociation rate constant	1 × 10^-3^	s^-1^	VEGF
*k_off,v164,N, _k_off,R1,N_*	V_164_N, R1N complex dissociation rate constant	1 × 10^-3^	s^-1^	VEGF
*k_on,v164,N_*	V_164 _N complex association rate constant	3.2 × 10^9^	pmol^-1 ^μm^3 ^s^-1^	VEGF
*k_on,R1,N_*	R1N association rate constant	1 × 10^10^	pmol^-1 ^μm^2 ^s^-1^	VEGF
*k_on,V164R2,N_*	V_164 _R_2_·N complex association rate constant	3.1 × 10^9^	pmol^-1 ^μm^2 ^s^-1^	VEGF
*k_on,V164N,R2_*	V_164 _N·R_2 _complex association rate constant	1 × 10^10^	pmol^-1 ^μm^2 ^s^-1^	VEGF
*S_V120,b_*	V_120 _basal secretion rate	0.17	fmol(L tissue)^-1^s^-1^	VEGF
*S_V164,b_*	V_164 _basal secretion rate	1.97	fmol(L tissue)^-1^s^-1^	VEGF
*B_tip-prolif_*	Boolean value of whether tip cells proliferate	No	-	Cell
*B_stalk-prolif_*	Boolean value of whether stalk cells proliferate	Yes	-	Cell
*B_tip-branch_*	Boolean value of whether tip cells branch	No	-	Cell
*B_stalk-branch_*	Boolean value of whether stalk cells branch	No	-	Cell
*B_tip-elongation_*	Boolean value of whether tip cell elongate	No	-	Cell
*B_stalk-elongation_*	Boolean value of whether stalk cells elongate	Yes	-	Cell

* Abbreviations: EBM, Endothelial Basement Membrane; MBM, Myocyte Basement Membrane; V_120/164_, VEGF_120/164_; N: Neuropilin 1. Note other agent-based rules and the parameters in Cell module can be found in [33].

Oxygen consumption rate, *M*_*c*_, increases to 2- to 50-fold the basal rate during exercise [68]. Previous models simulated the system under 6- and 12-fold oxygen consumption [22,61]. Here we used oxygen consumption of 9-fold the basal rate, corresponding to moderate exercise intensity conditions. These moderate exercise conditions are typical of experimental rat (mouse) aerobic treadmill exercise [69,70]. Another important parameter of the exercise environment is oxygen saturation of arterioles feeding into capillaries (*S_O2A_*). The model uses values of 0.6 and 0.3 for *S_O2A_*, corresponding to a normoxic environment and a low oxygen environment (hypoxic hypoxia). Hydrodynamic pressure drop between small arteries and venules (*ΔP*) is specified at 10 mmHg and inlet discharge hematocrit is 0.4, consistent with physiological observations [41,71]. For exercise-induced angiogenesis, experimental observations show that VEGF mRNA expression is elevated significantly within 1 hour post-exercise, and it remains elevated until returning to basal levels after 8 to 24 hours [69,70,72-74]. Due to the lack of experimental measurement of VEGF protein secretion rate from the myofibers *in vivo*, we assume that VEGF protein expression follows the similar time course as mRNA. Thus, we consider VEGF secretion as a step function starting at high levels at the onset of the post-exercise period, remaining constant for an 8-hour interval, and finally ending after 8 hours. We simulate the system for eight hours as the period of capillary growth; beyond this time elevated VEGF secretion ceases and the stimulus to sprout declines.

**Figure 3:**
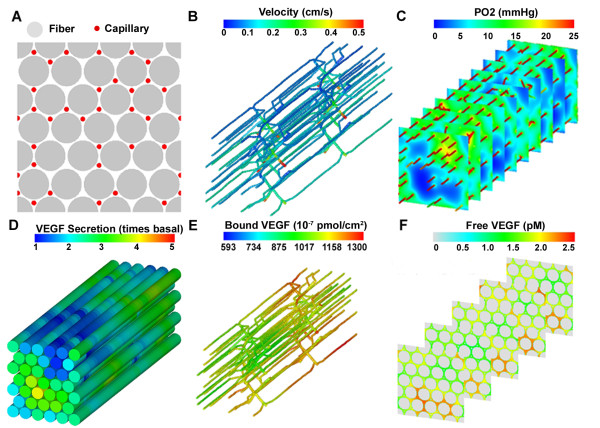
**3D simulation of blood flow, oxygen, and VEGF distribution during the single-bout exercise**: **A) **2D cross section of skeletal muscle: gray circles represent fibers and red circles represent capillaries; **B) **Blood flow velocity distribution in skeletal muscle microvascular network; **C) **Oxygen distribution throughout the tissue; **D) **VEGF secretion level along the muscle fibers; **E) **total VEGFR-bound VEGF distribution (including both 120 and 164 isoforms) on vascular surface; and **F) **free VEGF concentration distribution in interstitial space.

Using skeletal muscle geometry (Figure 3A) the blood flow simulation results (Figure 3B) show heterogeneous distribution of blood flow velocity in the microcirculatory network, with variation in velocity between blood vessels across the x-y plane. The mean flow velocity of all capillaries in the network is 0.11 cm/s and total blood flow is 227.82 ml·(100 g tissue)^-1 ^min^-1^, consistent with experimental measurements [75-77]. EDL tissue displays oxygen spatial heterogeneity not only in the x-y plane, but also in the z-direction, as shown in the 3D oxygen distribution plot (Figure 3C), generated from the Oxygen module simulation results. Most of the hypoxic tissue space has few nearby capillaries, confirming that the oxygen tension gradient and distribution is dependent on capillary spatial positions in the skeletal muscle space. Calculating all oxygen tensions in the tissue, we obtained an average tissue *P_O2 _*of 13.23 mmHg with a coefficient of variation 0.28.

Using oxygen tension distribution as input to the VEGF simulation parsed by the integration core package, we calculated VEGF secretion rate (Figure 3D). The average VEGF secretion level is 2.12-fold with a coefficient of variation of 0.32. The increase in VEGF secretion levels results in the increase of soluble VEGF concentration in tissue interstitial space (average free VEGF concentration is 1.9 pM). Simulation results from the VEGF module (Figure 3E) show that VEGF gradients in the longitudinal direction (along the fibers) are much smaller than in the cross-sectional planes, as indicated by their mean values (0.56% and 3.93% change in VEGF concentration over 10 microns, respectively). Similarly, VEGF binding to the endothelial surface receptor VEGFR2 is non-uniform across the vascular network (Figure 3F). The mean surface concentration of total receptor-bound VEGF is 1023 × 10^-7 ^pmol/μm^2 ^(coefficient of variation of 0.20), of which 8% is VEGF_120 _and 92% is VEGF_164_; NRP1-facilitated VEGF_120_-VEGFR1 and VEGF_164_-VEGFR2 complexes contribute 4.69% and 67.09% of the total receptor complexes, respectively.

We simulated the capillary activation process using our modified *in vivo *cell model. The precondition for an endothelial cell to be considered as activated is that any of its cell segments is exposed to a VEGF concentration greater than a specified VEGF activation threshold. A random selection algorithm is used to determine the sprouting position in the activated endothelial cells and the number of activated endothelial cells is also constrained by the allowed maximum number of activated tip cells per capillary (5 cells per capillary) and the minimum distance between tip cells (40 μm). These rules allow us to define activated tip cell and cell segments as the sprouting point for new capillary sprouts. Using a VEGF activation threshold of 2.0 pM, 2% percent of cells are activated and these cells initiate the sprouting process.

**Figure 4:**
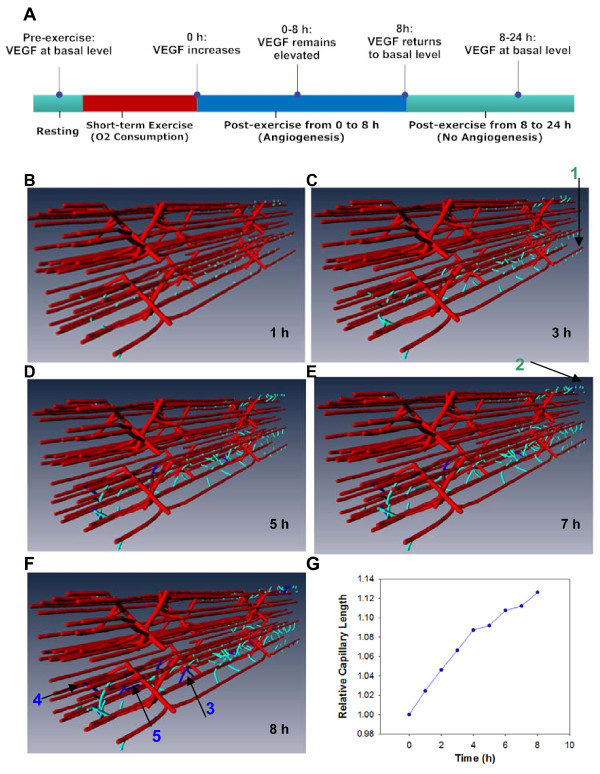
**3D simulation of capillary network growth during the single-bout exercise**. **A) **Time course of capillary growth was simulated based on the timeline scheme for exercise-induced angiogenesis. The simulation leads to new capillary network at: **B) **1 h post-exercise; **C) **3 h; **D) **5 h; **E) **7 h; **F**) 8 h. When new capillary segments grow out of boundaries (Arrow 1), capillary will grow from corresponding periodic boundaries (Arrow 2). Arrows 3-5 refer to the capillary anastomoses during growth. Vessels colored in red represent the pre-existing microvascular vessels, ones in light blue are the newly-developed blind-ended capillaries, and ones in dark blue are new capillary anastomoses. **G) **Relative capillary length change vs time.

We next simulate cellular sprouting and predict dynamic development of nascent vessels during 8 hours post-exercise, as defined in the timeline scheme in Figure 4A. Figure 4B-F shows new vascular network formation at 1, 3, 5, 7 and 8 hours following exercise. One of the assumptions in the model is that new capillary formation does not affect muscle fiber oxygenation, VEGF secretion or VEGF internalization, due to the slow capillary maturation process. Thus, a feedback loop is not implemented for a single bout of exercise, and a static VEGF profile is used as input for the cell model. This is a condition that can be relaxed in future models. We also assume periodic conditions for capillary growth, i.e., when capillary vessel sprouts encounter the boundary, they continue to grow beyond the boundary (e.g. arrow 1 at Figure 4C) and are represented by a corresponding capillary growing from the opposite face (e.g. arrow 2 at Figure 4E). As shown in Figure 4D-F, capillary sprouts on the same capillary initially migrate in a similar direction, while at a later time, they may form anastomoses with adjacent vasculature while migrating (arrows 3, 4, 5). Using parameter values listed in Table 1, capillary proliferation and migration rules defined in the cell module result in a 12.6% increase in total capillary length compared to the initial length before exercise (Figure 4F). However, for the conditions of a single bout of exercise considered in this study most of these new sprouts are likely to retract; sustained exercise conditions with formation of multiple functional anastomoses will be considered in subsequent studies.

### Sensitivity analysis of cellular parameters to define viable parameter space

**Figure 5:**
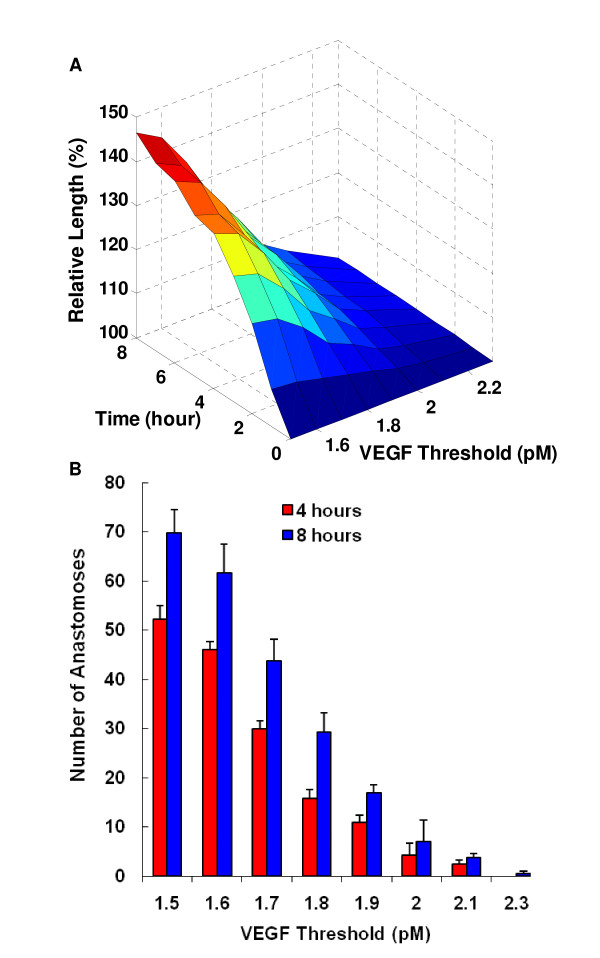
**Sensitivity of the capillary length and the number of anastomoses formed with respect to VEGF threshold for EC activation**. **A) **Relative capillary length as a function of time and VEGF threshold; **B) **Number of anastomoses formed as a function of VEGF threshold at 4 h and 8 h. Simulation sample size is five for each VEGF threshold at a given time.

We further explore the effect of parameters defined in the multiscale model of capillary growth. For this purpose, all biochemical, biophysical, and physiological parameters are extracted from each module and are deposited in a parameter database file. We show an example of how endothelial cell activation affects the capillary growth pattern and growth rate (two key variables related to capillary growth). We vary the threshold level of VEGF for endothelial cell activation within a range of 1.5 to 2.3 pM and this change significantly affects the capillary growth rate. Figure 5A shows that above a VEGF threshold of 2.3 pM, none of the endothelial cells are activated and the capillary network has no growth. Over the range of 2.3-1.8 pM, capillary growth rate increases slightly as the threshold value decreases. Below 1.8 pM, capillary growth increases dramatically since a large number of capillaries are activated. The response to these threshold levels depends on the mean concentration of VEGF in the tissue (here, 1.9 pM).

The number of anastomoses is another important variable that indicates vascular adaptation of the microvasculature since the increase of blood flow results from these newly-formed vessel loops (and not from blind-ended vessels). We show in Figure 5B that the number of anastomoses increases in a similar fashion when threshold values decrease from 2.3 pM to 1.5 pM. Interestingly, more than half of the anastomoses are predicted to form during the first 4 hours.

### Simulation of exercise-induced angiogenesis under different conditions

**Figure 6:**
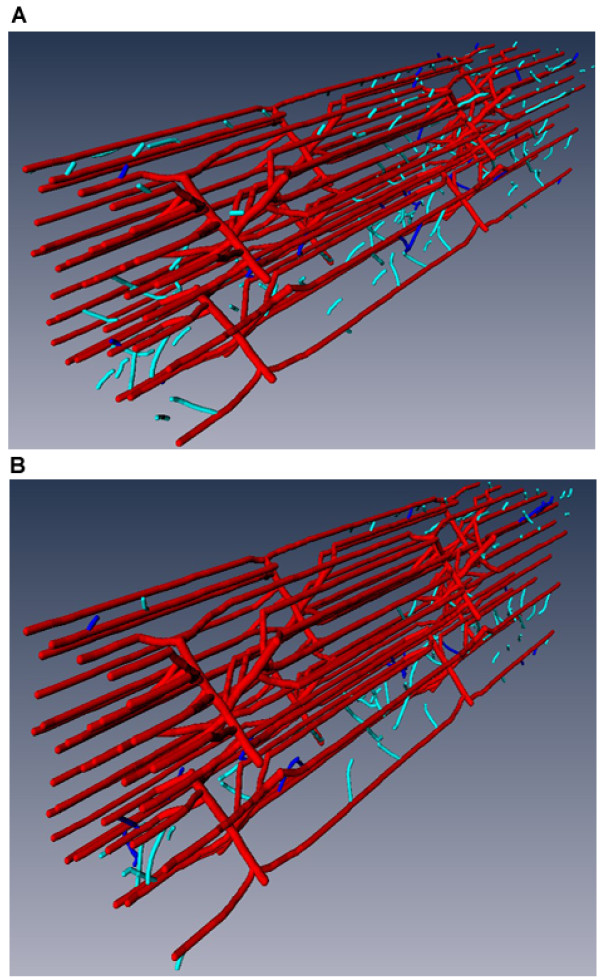
**Angiogenesis pattern during different exercise conditions at 8 h post-exercise**. **A) **Oxygen consumption at 9-fold the basal level under hypoxic hypoxia conditions (*S_O2A _*= 0.3); **B) **Oxygen consumption at 9-fold the basal level under normoxic conditions (*S_O2A _*= 0.6).

Varying experiment-based parameters defined in the Oxygen modules, we can simulate vessel growth in skeletal muscle during different exercise conditions. For example, different oxygen consumption rates, *M_C, _*correspond to different exercise intensities and arterial oxygen saturation values *S_O2A _*are associated with different available oxygen levels. We compare capillary growth patterns between three exercise conditions: i) *M_C _*= 1 × 10^-3 ^ml O_2_/ml tissue/s and *S_O2A _*= 0.6 (light intensity exercise in normoxia; results not shown); ii) *M_C _*= 1.5 × 10^-3 ^and *S_O2A _*= 0.6 (moderate intensity exercise in normoxia; Figure 6B and 4F); iii) *M_C _*= 1.5 × 10^-3 ^and *S_O2A _*= 0.3 (moderate intensity exercise in hypoxic hypoxia; Figure 6A). Simulation results show that during low-intensity exercise (6-fold basal level oxygen consumption rate) with normoxic conditions, the capillary network will not change (case i). Compared to exercise condition of a 9-fold basal oxygen consumption rate in normoxia (case ii), exercise under hypoxic hypoxia conditions at a 9-fold basal oxygen consumption rate further increases capillary growth to 125% (case iii).

## Discussion and Conclusions

Using a module-based computational platform, we connected four modules, each of which describes different aspects of the angiogenesis process (i.e, blood flow, oxygen distribution, VEGF distribution, and capillary growth). This architecture allowed the individual modules to remain in their native language and required rewriting only of the input/output routines to handle the passing of common data between the submodules and the integrator. This significantly reduced the time required to integrate the available modules. JNI plugins are used to link the codes written in different languages, and they allow ready communication between the core package and each module. As a validation of our simulation strategy and integration package, we have conducted a series of computational experiments to simulate dynamic activity-induced angiogenesis during a single bout of exercise, using rat EDL skeletal muscle. We illustrated how the computational design of a core controller module is beneficial to integrating different aspects of biological knowledge with a variety of numerical simulation schemes: i) sequential integration; ii) sensitivity analysis; and iii) simulation of various exercise conditions.

Object-oriented programming (OOP) is attracting more modelers' attention and has been increasingly used in biomedical computational projects [56,57,78]. We applied OOP concepts to describe a complex biological system which encompasses biological components and processes from the molecular level to the tissue level. With our model architecture, we also depicted the hierarchical relationships among the different classes abstracted from such systems. For the core package, Java was used since it has a natural object-oriented design and method inheritance structure that is intuitively appealing to describe the biological system.

Our approach is complementary to other ongoing projects in multiscale modeling fields, e.g. CompuCell [79], the Multiscale Systems Immunology (MSI) simulation framework [80] and Virtual Cell [81]. These projects share the same objective: the development of multiscale modeling software or libraries with a versatile user interface. CompuCell implements cellular Potts models to simulate cell dynamics, MSI uses agent-based models and Virtual Cell uses differential equations. While we did not attempt to provide standards or software for the angiogenesis models, we chose to focus on the extensibility and generality of an integration methodology that allows additional individual modules to be integrated at any time, and does not impose constraints on the modeling techniques employed.

As a starting point, current study shows an example of the simulation by integrating four modules. We used existing models for the first modules to be able to compare the results of the integrated model to published results of each submodule. This computational paradigm will allow us to incorporate additional modules developed in the laboratory, e.g., MMP [23,24], HIF1α [82] and FGF2 [18], using an SBML/CellML-based open source simulation packages to represent the molecular interaction networks. In addition, this platform can incorporate other relevant published models from the literature or public model repositories, e.g. CellML Models Repository http://models.cellml.org/ and BioModels Database http://www.ebi.ac.uk/biomodels-main/. It is also feasible to improve on or add to the individual modules currently comprising the integrated model. For example, the geometry used by the modules can be updated to that of a different muscle type without rewriting the other module codes; or more detailed information such as the fiber type of each muscle fiber can be included as an additional data notation, with corresponding changes in local oxygen consumption and O_2 _diffusion coefficient and solubility.

Correspondingly, the modeling controller module will be further expanded to include the classes representing other components, which may be relevant to skeletal muscle angiogenesis when new biological information and models are available. In the long run, definition of unified XML-based notation to describe structure and spatial information of physiological systems, ordinary/partial differential equations, agent-based rules and system parameters will make each module more portable and more easily integrated. Progress has been initiated in this direction such as insilicoML [83]. In addition, development of the ontology for the field of angiogenesis modeling will also be beneficial to the sharing, usage and integration of different modules, similar to gene ontology [84], and the open biological and biomedical ontologies [85]. These ongoing and future developments should contribute to the overall efforts of the Systems Biology and Physiome Projects [46,86], and could be applied to cardiovascular disorders such as coronary and peripheral artery diseases [87].

Along with this framework for modeling the system, there is now the opportunity to include other useful functionality such as local and global parameter sensitivity modules, or parameter optimization modules. While local parameter sensitivity calculations can be performed with the current model, the computational expense of the model (in particular the PDE solver) is such that parameter estimation would be very slow. We aim to use cluster computing in order to permit these activities in the future.

In summary, we have developed the computational methodology capable of the integration of biologically- and computationally-heterogeneous modules and this systems biology strategy can be applied to larger scale integration of computational models of angiogenesis in skeletal muscle.

## Competing interests

The authors declare that they have no competing interests.

## Authors' contributions

Conceived and designed the simulations: GL AAQ PV FMG ASP; Performed the simulations: GL; Analyzed the data: GL AAQ PV FMG ASP; Wrote the paper: GL AAQ PV FMG ASP. All authors read and approved the final manuscript.

## Supplementary Material

Additional file 1 **Modified empirical equations for the relationship between *P_O2 _*and VEGF secretion rate**. Click here for file
