# Supplementary material for: Module-based multiscale simulation of angiogenesis in skeletal muscle
Source: Theor Biol Med Model. 2011 Apr 4;8:6. doi: 10.1186/1742-4682-8-6 (PMC3079676; doi:10.1186/1742-4682-8-6)
Supplement: Additional file 1 — Modified empirical equations for the relationship between PO2 and VEGF secretion rate. [file 1742-4682-8-6-S1.DOC]

Modified empirical equation of PO2- VEGF secretion relationship

Our previous empirical equation describing the relationship between PO_2_ and VEGF secretion is given in Eqn. S1. The construction of this PO2-VEGF relationship was based on the assumption that VEGF regulation through oxygen sensing was conducted solely through the canonical HIF pathway, with HIF-1α acting as the oxygen sensor.

$S=\left\{\begin{matrix} S_{0},\text{ PO}_{2}\geq20\text{ }mmHg \\ S_{0}\times\left[1+5\times\left(\frac{20-\text{PO}_{2}}{19}\right)^{3}\right] \\ 6S_{0},\text{PO}_{2}<1\text{ }mmHg \end{matrix}\right.$ **(S1)**

To incorporate effect of PGC-1α into this relationship, the following model is proposed:

$S_{VEF}=\left\{\begin{matrix} S_{B,VEGF},\text{ PO}_{2}\geq20\text{ }mmHg \\ S_{B,VEGF}\times\left[1+(\alpha_{HIF}^{\max}-1)\times\left(\frac{20-\text{PO}_{2}}{19}\right) ^{3}\right] \\ S_{B,VEGF}\times\alpha_{HIF}^{\max},\text{PO}_{2}<1\text{ }mmHg \end{matrix}\right.$ (S2)

$S_{B,VEGF}=S_{0}\times[A(\frac{[\text{PGC-1α}]^{n}}{k_{h}+[\text{PGC-1α}]^{n}})+B]$ (S3)

Where *S_B,VEGF_* is defined as basal VEGF secretion rate at normoxic [HIF1-α] level (i.e, it may change with the variation of PGC-1α protein concentration, and [PGC1-$\text{α}$] is the normalized PGC-1α concentration relative to normoxia PGC-1α expression for wild type skeletal muscle. A sigmoidal form is assumed for *S_B,,VEGF_* as defined in S3. It includes two terms. The first term refers to effect of PGC-1α level on VEGF secretion, written as a Hill equation, where n is hill constant and *k*_h_ is velocity constant. Second term includes other factors which may also contribute to VEGF secretion, approximated as a constant *B*. *S_0_* is defined as basal VEGF secretion rate at normoxia [HIF1-α] and [PGC-1$\text{α}$].

There is a lack of empirical data available to describe the PO_2_ - PGC1-α relationship. Thus, an empirical equation is assumed for normalized [PGC1-α] expression in wild type, knockout and overexpressed muscle, written as below:

**(S4)**

**(S5)**

**(S6)**

where [PGC-1α]_WT_, [PGC-1α]_KO_ and [PGC-1α]_OE_ represent the normalized PGC-1α concentration in wild type, a PGC-1α-knockout and a PGC-1α -overexpression model respectively. A logistics curve is assumed to represent the relationship between [PGC1-α] and PO_2_. [PGC1-α]_KO_ is assumed to be invariantly zero throughout and [PGC1-α]_OE_ takes on a constant value that correlates with experimental values.

Compilation of Experimental Data

We searched thoroughly the literatures related to skeletal muscle [VEGF] expression, and table S1 below shows the relevant experimental values we used to solve for Eqn. S2-6:

Table S1

| **PGC-1α KO** | **PGC-1α**  **OE** | **HIF-1α**  **KO** | **Hypoxia** | **[PGC1-α]** | **S** | **Refs** |
| --- | --- | --- | --- | --- | --- | --- |
| No | No | No | No | 1 | 1 | N/A |
| No | No | No | Yes | 3 | 5 | [1, 2] |
| Yes | No | No | No | 0 | 0.35 | [3] |
| No | Yes | No | No | 10 | 2 | [1, 2] |
| No | Yes | No | Yes | 10 | 8 | [1] |
| No | No | Yes | No | 1 | 1 | [4] |
| No | No | Yes | Yes | ND | 1.7 | [4] |

Parameter Identification

A system of equations was formed using the data in Table S1 and Equations 1-5. Solving it yields the following values for the unknown parameters:

**Table S2: Optimized Parameter values**

| **A** | **B** | **E_max_** | **n** | ***k_h_*** | $\alpha_{HIF}^{\max}$ |  |
| --- | --- | --- | --- | --- | --- | --- |
| 2.316 | 0.35 | 10 | 1.086 | 2.5641 | 3 | 3 |

Plot of VEGF-O2 under various conditions

Using Eqn. 2 -5 and optimized parameter values, we simulated how VEGF secretion rate varies with tissue oxygen tensions under a series of animal models, such as knockout, overexpression, normal state of PGC1-α, and knockout or overexpressed HIF (Figure S1 below). In the main manuscript, we use a curve for wild type to represent the relationship between VEGF secretion rate and oxygen tension.

Figure S1

References:

1. O'Hagan KA, Cocchiglia S, Zhdanov AV, Tambuwala MM, Cummins EP, Monfared M, Agbor TA, Garvey JF, Papkovsky DB, Taylor CT, Allan BB: **PGC-1alpha is coupled to HIF-1alpha-dependent gene expression by increasing mitochondrial oxygen consumption in skeletal muscle cells.** *Proc Natl Acad Sci U S A* 2009, **106:**2188-2193.

2. Arany Z, Foo SY, Ma Y, Ruas JL, Bommi-Reddy A, Girnun G, Cooper M, Laznik D, Chinsomboon J, Rangwala SM, et al: **HIF-independent regulation of VEGF and angiogenesis by the transcriptional coactivator PGC-1alpha.** *Nature* 2008, **451:**1008-1012.

3. Leick L, Hellsten Y, Fentz J, Lyngby SS, Wojtaszewski JFP, Hidalgo J, Pilegaard H: **PGC-1 alpha mediates exercise-induced skeletal muscle VEGF expression in mice.** *American Journal of Physiology-Endocrinology and Metabolism* 2009, **297:**E92-E103.

4. Mason SD, Howlett RA, Kim MJ, Olfert IM, Hogan MC, McNulty W, Hickey RP, Wagner PD, Kahn CR, Giordano FJ, Johnson RS: **Loss of Skeletal Muscle HIF-1伪 Results in Altered Exercise Endurance.** *PLoS Biol* 2004, **2:**e288.
